# Hallucination in a Seizure Patient Using Levetiracetam: A Case Report

**DOI:** 10.1155/2012/706243

**Published:** 2012-07-19

**Authors:** D. R. Shakya, A. Dutta, R. Gautam

**Affiliations:** Department of Psychiatry, B. P. Koirala Institute of Health Sciences, 56700 Dharan, Nepal

## Abstract

Levetiracetam, a relatively new antiepileptic drug (AED), is used mainly as adjuvant and less as monotherapy of seizure. Though rare, Levetiracetam is reported to induce hallucination. To highlight the potential of this adverse drug event, we report a seizure-case that had auditory hallucination with Levetiracetam. A 32-year lady had 7-year history of unresponsive spells which increased in the last year, also occurred while asleep and were diagnosed as “generalized seizure” with video-EEG. With gradual optimization of Levetiracetam to 2250 mg, she continuously heard distressing sound of saw cutting wooden blocks. After 3-day continuous auditory hallucination, Levetiracetam had to be changed to sodium valproate.

## 1. Introduction 

Levetiracetam is the (S)-enantiomer of the ethyl analog of the nootropic agent “Piracetam.” Its structure is unrelated to other anticonvulsants. It was FDA approved in November 1999 for adjunctive therapy of partial seizures of adult. Levetiracetam is currently indicated also as adjunctive treatment of primary generalized tonic-clonic and myoclonic seizures; especially when refractory [1]. Its antimanic [2] and other psychotropic uses need further studies [3].

Though generally well tolerated, it may cause some adverse reactions. Commonly observed neurological problems include asthenia, ataxia, diplopia, dizziness, dysarthria, fatigue, headache, light-headedness, nystagmus, paresthesias, somnolence and tremor. They usually are either dose related or transient. Behavioral effects include agitation, anxiety, depression, emotional lability, hostility, nervousness, and psychosis which are less clearly related to drug, dose or tolerance [1, 4, 5]. Though rare, hallucinations have been reported since early years of its introduction [5]. Here is a seizure case who experienced continuous distressing auditory hallucination with 2250 mg of Levetiracetam that needed to be stopped, and later Sodium valproate had to be started for seizure control.

## 2. Case Report 

A 32-year-old married female, educated up to intermediate of arts, employed as a primary school teacher, presented to psychiatry outpatient clinic of a teaching hospital with complaint of frequent unresponsive spells for last 7 years. The first attack occurred during the third trimester of her first pregnancy and subsequently had 3-4 episodes in 2-3 days. The unresponsive spells were characterized by stretching and twisting of bilateral upper and lower limbs for 10–15 minutes, associated with extension of neck, closing of eyes and spontaneous gain of consciousness. The spells occurred only in presence of family members, not during sleep. There was no history of physical injury, tongue bite, and incontinence. With Phenytoin 300 mg started from a private practitioner, the spells were still occurring 2-3 times in 6 months and hence, she stopped medicine by herself. Surprisingly, no such spells were noticed for next 2 years. She had similar 3-4 episodes again during the third trimester of the second pregnancy 5 years back. She did not receive any treatment then. During both the first and second pregnancies/deliveries, she did not have constitutional symptoms or infections, antenatal checkups were done with routine investigations which were reported verbally by patient/family to be normal and deliveries were uneventful. There were no such episodes for next 4 years. 

Since the last year, the unresponsive spells were occurring again but were different in presentation and characteristics from previous spells. These spells lasted for 3–7 minutes and occurred also during sleep. Her husband noticed that patient did not get up even on vigorous shaking during such a spell whereas she would be easily arousable from normal sleep. During these spells, she would remain unresponsive and her body would be lax without movement of any type, unlike before. She would feel weak or experience heaviness of head on subsequent day for variable hours. There was no history of other sleep-related problems. Such episodes were occurring more frequently in last 3-4 months. With medical consultation and video EEG, an impression of “generalized epileptiform discharges” was made, and she was advised Levetiracetam 750 mg/day in a seizure specialty hospital of a private setup. It was gradually increased up to the dose of 2250 mg over a period of 3 weeks (with the target dose 3000 mg). 

After about 24 hours of consumption of 2250 mg in the 3rd week of initiation of Levetiracetam, the patient started hearing the sound of saw cutting wooden blocks which other people nearby did not hear. The sound was high-volume, continuous, and distressing. It was so intolerable to the patient that she would be restless and felt as if her head was tearing apart. She tried to cover her ears but it did not decrease the intensity of the sound and the sound would begin or terminate for a while in between, with no control of her over it. The sounds were heard equally in both ears in a full conscious state. Besides this hallucination, no other psychotic or mood symptoms were noticed. After 3 days of such a continuous distressing experience, the same treating physician was consulted who instructed to decrease Levetiracetam to 1500 mg. The hallucination disappeared but the seizure continued with same frequency. Because of such a distressing experience and continuing seizure spells, the patient was brought to psychiatry outpatient clinic of B P Koirala Institute of Health Sciences, a teaching hospital in eastern Nepal. 

There was no report of perceived stressor preceding current exacerbations. She did not have past history of any significant head injury, CNS infection, or psychiatric including psychotic disorder including problematic substance use. They did not remember any close blood relatives suffering from similar illness or other mental disorders. General and systemic examination then, including neurological examination revealed no abnormality. Her weight was 55 kg and height 5′4′′. The MRI scan of brain (done in the private hospital and the repeat assessment) revealed normal finding. After a detail assessment, an impression of “seizure disorder with history suggestive of Levetiracetam-induced hallucination” was made. Sodium valproate was advised with the target dose of 1000 mg with which she was seizure free till the latest followups of its 9 month treatment.

## 3. Discussion 

Seizure disorder is the main indication of Levetiracetam. Levetiracetam is an established second generation antiepileptic drug (AED) and currently is indicated as adjunctive treatment of: (1) partial seizures in adult and children of 4 years or more with epilepsy, (2) myoclonic seizures in adults and adolescents of 12 years or more with juvenile myoclonic seizure, and (3) primary generalized tonic-clonic seizures in adults and children 6 years and more with idiopathic generalized epilepsy [1]. It is also approved as monotherapy for partial onset seizures with or without secondary generalization [6]. Levetiracetam, a Piracetam analog, that is, “(−)-(S)-*α*-ethyl-2-oxo-1-pyrrolidine acetamide” has a novel structure and unique mechanism of action. It binds to synaptic vesicle protein 2A, inhibiting calcium release from intra-neuronal stores, opposing the activity of negative modulators of GABA- and glycin-gated currents and inhibiting excessive synchronized activity between neurons. It also inhibits N-type calcium channels [1]. Levetiracetam is rapidly and completely absorbed through GIT, resulting in a high oral bioavailability. It is mainly excreted unchanged through renal elimination. It has no CYP450 isoenzyme-inducing potential and is not associated with clinically significant pharmacokinetic interactions with other drugs, including other AEDs including Sodium valproate [1]. Its efficacy in controlling seizures has been ascertained in numerous randomized, double-blind, controlled, multicenter trials, mainly as adjunctive and relatively less as monotherapy [6]. In this reference case, monotherapy with Levetiracetam appears to be the target of the specialty hospital but later it had to be decreased, stopped, and changed to Sodium valproate because of intolerable and distressing hallucination and continuing seizure. 

The overall proportion of patients with at least one treatment-emergent adverse event was reported similar in Levetiracetam and placebo treatment groups in clinical trial studies (53–89% versus 53–92%) [7]. No relationship was shown between the dosage of Levetiracetam and incidence of behavioral adverse events in clinical trials [6, 7]. In our case however, the psychotic symptom, hallucination appeared at the dosage of 2250 mg of Levetiracetam which was not seen with the dose of 1500 mg. Most of these adverse events were mild to moderate in severity though there were reports of discontinuation of the AED because of adverse event [8–10]. Serious events were seen comparable in incidence to placebo (0–4.7% versus 1.0–2.7%) [7–10]. 

Levetiracetam is not associated with cognitive impairment or weight gain as other AEDs but is associated with behavioral adverse effects in some patients (1–10%) [4, 6]. The most common treatment-emergent adverse events with Levetiracetam are asthenia, somnolence, infection and dizziness in adults and somnolence, accidental injury, hostility, nervousness, and asthenia in children [4, 6, 11]. Behavioral problems include aggression, agitation, anger, anxiety, apathy, depersonalization, depression, emotional lability, hostility, and irritability, especially in the first month of use. Suicidal ideation and behavior is reported to increase with its use and hence, the Food and drug Administration (FDA) has announced warning of increased suicidal risk with this AED like other AEDs [1]. It is however, noteworthy that seizure in itself is associated remarkably with psychiatric symptoms and disorders [12]. 

Behavioral symptoms of hallucinations have been reported with the use of this agent from early years of its use [4, 6]. Hallucination of not only auditory but also other modalities has been reported, for example, visual [13]. This particularly was mentioned in the 8th edition of Comprehensive Textbook of Psychiatry (2005) [5] which was less described in the latest 9th edition (2009) [1] probably because it is rare and was not reported/seen in subsequent years. However, based on the method of Naranjo et al. for estimating the probability of this symptom of hallucination being adverse drug reaction, the adverse event appear after it was given (2 points), the symptom improved when it was decreased/discontinued (1 point) and no alternative causes could be ascertained for hallucination (2 points) making total points of at least 5 out of 13 (i.e., possible to probable ADR) [14]. Hence, this case report indicates that though rare, hallucination does occur as a treatment-emergent adverse event with Levetiracetam. And, this fact needs to be kept in mind while using it.
